# Canadian children's and youth's pedometer-determined steps/day, parent-reported TV watching time, and overweight/obesity: The CANPLAY Surveillance Study

**DOI:** 10.1186/1479-5868-8-66

**Published:** 2011-06-25

**Authors:** Catrine Tudor-Locke, Cora L Craig, Christine Cameron, Joseph M Griffiths

**Affiliations:** 1 Walking Behaviour Laboratory, Pennington Biomedical Research Center, Baton Rouge, LA 70808, USA; 2 Canadian Fitness and Lifestyle Research Institute, Ottawa, ON K2P 0J2, Canada; 3 School of Public Health, University of Sydney, Sydney, Australia

## Abstract

**Background:**

This study examines associations between pedometer-determined steps/day and parent-reported child's Body Mass Index (BMI) and time typically spent watching television between school and dinner.

**Methods:**

Young people (aged 5-19 years) were recruited through their parents by random digit dialling and mailed a data collection package. Information on height and weight and time spent watching television between school and dinner on a typical school day was collected from parents. In total, 5949 boys and 5709 girls reported daily steps. BMI was categorized as overweight or obese using Cole's cut points. Participants wore pedometers for 7 days and logged daily steps. The odds of being overweight and obese by steps/day and parent-reported time spent television watching were estimated using logistic regression for complex samples.

**Results:**

Girls had a lower median steps/day (10682 versus 11059 for boys) and also a narrower variation in steps/day (interquartile range, 4410 versus 5309 for boys). 11% of children aged 5-19 years were classified as obese; 17% of boys and girls were overweight. Both boys and girls watched, on average, < 40 minutes of television between school and dinner on school days. Adjusting for child's age and sex and parental education, the odds of a child being obese decreased by 20% for every extra 3000 steps/day and increased by 21% for every 30 minutes of television watching. There was no association of being overweight with steps/day, however the odds of being overweight increased by 8% for every 30 minutes of additional time spent watching television between school and dinner on a typical school day.

**Discussion:**

Television viewing is the more prominent factor in terms of predicting overweight, and it contributes to obesity, but steps/day attenuates the association between television viewing and obesity, and therefore can be considered protective against obesity. In addition to replacing opportunities for active alternative behaviours, exposure to television might also impact body weight by promoting excess energy intake.

**Conclusions:**

In this large nationally representative sample, pedometer-determined steps/day was associated with reduced odds of being obese (but not overweight) whereas each parent-reported hour spent watching television between school and dinner increased the odds of both overweight and obesity.

## Background

Although increasing trends in childhood overweight and obesity display evidence of abatement in some regions of the world [1], the same cannot be said for the U.S. [2] or Canada [3]. Case in point, levels of obesity have significantly increased over 15 years for Canadian boys (2 to 10%) and girls (2 to 9%) [4]. While changes in dietary energy intakes cannot be ruled out, another potential culprit that likely contributes to this unfortunate state of affairs is reduced physical activity. In particular, there have been noticeable secular transitions in parents' well-meaning concerns for safety that sequester children inside [5,6], and a persistent predilection for (and acceptance of) passive recreational pursuits including sustained record levels of television viewing behaviours, despite competition from other electronic media [7].

Objective assessment of physical activity using accelerometers and pedometers has become more common practice in the study of childhood overweight and obesity. For example, pedometer-determined steps/day were generally significantly higher for normal weight children vs. overweight/obese children for each sex-age group from 6-12 years of age in an international sample [8]. Although accelerometers do provide important information on physical activity intensity (an important component of most public health recommendations), pedometers are generally considered more feasible in terms of cost and ease of use [9], and therefore are a more practical alternative for population level strategies, including large nationally representative surveillance studies.

No body-worn objective measure of physical activity is capable of capturing type of activity, however, and this includes television watching behaviours. Yet such behaviours have been linked to increased risk of overweight/obesity in Canadian children [10] and U.S. children and youth [11]. A 2007 review concluded that evidence of a relationship between television watching and physical activity is equivocal in youth [12], although a more consistent relationship has been found for sedentary behavior after school among adolescents [13]. More recently, increased amounts of television watching have been associated with decreased pedometer-determined steps/day [14]. Specifically, Laurson and colleagues [14] recently reported that those who did not meet pedometer-determined cut points or screen time recommendations (i.e., ≤ 2 hours/day) in a sample of 709 children aged 7-12 years were 3-4 times more likely to be overweight compared to those who met both.

Canadian Physical Activity Levels Among Youth (CANPLAY) is a pedometer-based nationally representative surveillance study conducted with children and youth (aged 5 to 19 years; representing roughly 12000 families) from across all areas of Canada between September 20, 2005 and April 30, 2007. In total, 11669 young people complied with the pedometer protocol aspects of the study. Details of the data collection process and subsequent treatment have been previously published [15]. This study examines associations between pedometer-determined steps/day and parent-reported child's Body Mass Index (BMI) and time spent watching television between school and dinner on a typical school day.

## Methods

### Data Collection

The execution of the CANPLAY surveillance study was contracted by the Canadian Fitness and Lifestyle Research Institute to the Institute for Social Research (ISR) at York University. Households (14,858 households out of an estimated 20,802 eligible households) were initially contacted using random digit dialling [14]. Computer-assisted telephone interview (CATI) was then used to identify a randomly selected respondent who was at least 20 years of age and a parent or legal guardian of a child between 5 and 19 years of age living in the household. If the parent verbally agreed to their child's participation in the pedometer portion of the study (n = 19725 children), they were mailed a package that included a pedometer data self-collection kit. A more detailed explanation of the kit's contents, schedule of prompting to encourage data return, quality control treatments, and the full data collection process is provided elsewhere [15]. Briefly, parents were asked to have their children wear the pedometer for 7 consecutive days and to log daily steps. Logs were returned by 60% of those recruited (of whom about 95% complied with the 7 day protocol). Verbal informed consent was received during the recruitment interview for all participants followed by written assent on the log forms. The method and protocols were approved by the Human Participants Review Committee of York University.

Child's sex, age (in years), and responding parent's education level (< secondary education, secondary school graduation, or post-secondary training including college or university education) were gathered during the recruitment interview. Parents also provided an estimate of their child's height and weight in either metric or imperial units as preferred. Although most parents were able to supply an estimate of their child's weight (only 6.7% missing), a sizeable proportion could not do the same for their child's height (33.2% missing, valid n = 7495). The time that their child spent watching television between school and dinner on a typical school day was asked and recorded in hours and minutes. The specific time frame queried for this behaviour was based on a review that indicated that after school time was consistently related to physical inactivity in youth [13].

### Data Treatment

After converting any data expressed in imperial units to metric units, BMI was calculated as weight (kg)/height (m^2^). Overweight and obesity were categorized according to Cole's age- and sex-specific BMI cut-offs [16], which predict the World Health Organization (WHO)-defined adult cut-offs for overweight (> 25 and < 30 kg/m^2^) and obesity (≥ 30 kg/m^2^) [17].

Pedometer data below 1000 and above 30000 steps/day were truncated to those values [15]. Mean steps/day were computed averaging steps taken considering all available logged days across all days of the week and also restricted to only weekdays. We compared frequency distributions for boys' and girls' steps/day data rounded to the nearest 1000 steps/day increment. We also plotted age- and sex-specific mean steps/day (across all days) against expected (i.e., normative) values based on previously assembled international pedometer studies of free-living children and youth [18]. Means and 95% confidence intervals for steps/day and television watching time were calculated for child's age and sex, BMI-defined weight status, and responding parent's education.

We examined the distribution of pedometer-determined physical activity by sex among Canadian youth. Prevalence estimates and 95% confidence intervals were computed for age- and sex-specific steps/day. Differences in mean steps/day and television watching time between the full sample and participants with a valid BMI were examined separately for boys and girls using an independent t-test in each case. Since there were no significant differences, only the data for the full sample is presented herein.

We examined the relationship between pedometer-determined physical activity and parent-reported television watching between school and dinner by calculating sex-specific mean steps/day by television watching in 30 minute increments (from < 30 to 150+ minutes). An ANOVA was run to evaluate significant trends across incremental categories.

Logistic regression was employed to examine the associations between steps/day, parent-reported television watching time, and overweight and obesity status separately and combined (i.e., overweight/obese), controlling for child's sex and age, and parent's education level. The interaction between steps/day and television watching time was tested in each model. All estimates were computed using SPSS (Version 18) Complex Samples procedures accounting for the sample design and tested by applying sequential Bonferroni adjustments to the 95% confidence intervals.

## Results

### Pedometer-determined Physical Activity

**Figure 1:**
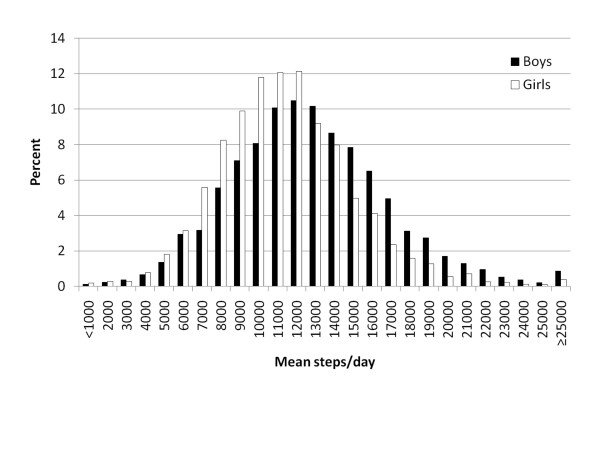
**Distribution of daily steps among Canadian boys and girls, 5-19 years of age**.

In total, 5949 boys and 5709 girls reported daily steps. As depicted in Figure 1, the frequency distribution of pedometer-determined steps/day was symmetrical for boys and girls, however girls had a lower median steps/day (10906 versus 11987 for boys) and also a narrower variation in steps/day (interquartile range, 4410 versus 5309 for boys). Mean steps/day were higher among boys and girls whose responding parent reported a university education, and lower among boys who were classified as obese (Table 1). Boys aged 14-19 years took fewer steps/day than younger age groups of boys. For girls, 5-9 year olds took the most steps/day, followed by 10-13 year olds, and finally 14-19 year olds took the fewest steps/day. These results were all significant based on interpretation of non-overlapping confidence intervals.

**Table 1:** Pedometer-determined steps/day and parent-reported television watching on a typical school day, Canadian boys and girls age 5-19 years

	Pedometer (weekly)	95% CI			Pedometer (week days)	95% CI			TV watching	95% CI		
	Mean Steps/day	Lower	Upper	N	Mean Steps/day	Lower	Upper	N	Mean Minutes	Lower	Upper	N
Boys												

5 to 9 years	12813	12586	13040	2094	13135	12905	13365	2092	35	32	37	2004
10 to 13 years	12845	12588	13103	1961	13341	13088	13594	1953	40	38	43	1883
14 to 19 years	11173	10908	11439	1894	11473	11212	11734	1890	43	40	47	1799

< Secondary	11528	10958	12099	447	11968	11381	12555	446	46	38	55	418
Secondary	11824	11498	12151	1275	12272	11932	12612	1270	42	38	46	1213
College, trades	12415	12134	12695	1903	12686	12421	12950	1900	40	37	44	1830
University	12508	12244	12772	2238	12891	12627	13155	2234	36	34	39	2174

Healthy weight	12235	12008	12463	2662	12591	12371	12812	2655	37	35	40	2562
Overweight	12162	11792	12532	825	12625	12243	13007	823	40	35	44	794
Obese	11518	11048	11987	543	11741	11257	12225	541	47	41	53	523

Girls												

5 to 9 years	11738	11529	11948	2029	11995	105	11790	12201	32	29	34	1963
10 to 13 years	11265	11040	11491	1917	11701	117	11472	11931	39	36	42	1844
14 to 19 years	9717	9497	9937	1763	10092	114	9868	10316	38	34	41	1675

< Secondary	9791	9319	10263	477	10229	242	9754	10704	47	40	55	450
Secondary	10565	10280	10851	1298	10917	141	10641	11193	38	35	42	1255
College, trades	11078	10816	11340	1778	11345	133	11084	11605	37	34	40	1723
University	11194	10975	11412	2086	11590	114	11366	11814	31	28	33	2019

Healthy weight	10859	10681	11036	2780	11212	92	11032	11393	34	32	36	2674
Overweight	10807	10411	11203	591	11248	200	10856	11641	42	36	47	570
Obese	10722	10131	11313	442	10975	277	10433	11517	45	38	51	426

**Figure 2:**
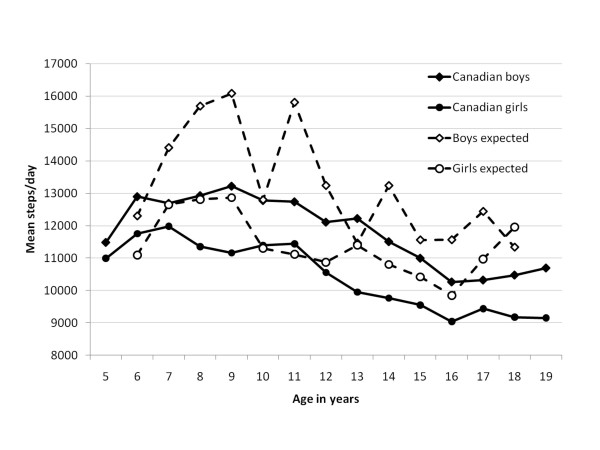
Sex- and age-specific median versus expected* pedometer-determined steps/day in Canadian boys and girls, 5-19 years of age. *from international studies of physical activity in free-living populations

Figure 2 shows that pedometer-determined steps/day taken by Canadian boys and girls were generally lower than those expected given published normative values [18]. This was most evident among boys younger than 13 years of age and girls younger than 10 years of age.

### Parent-reported Typical Television Watching Time Between School and Dinner

Parent-reported time spent watching television between school and dinner on a typical school day is summarized in Table 1. Both boys and girls watched, on average, < 40 minutes of television (mean = 39, 95% CI = 37-41 for boys; mean = 36, 95% CI = 34-37 for girls). Television viewing time increased by age group from 35 (among boys 5 to 9) to 43 minutes (among boys 14-19 years) and from 32 to 38 minutes respectively among similarly aged girls. Time spent watching television was lower among children of university educated parents than among children whose parent had less than secondary education; however, this was significant only for girls (31 minutes for university education, 47 minutes for < secondary education). Parent-reported television watching time between school and dinner increased by BMI-defined weight status.

### Relationship Between Pedometer-determined Steps/day and Parent-reported Television Watching Time Between School and Dinner

**Figure 3:**
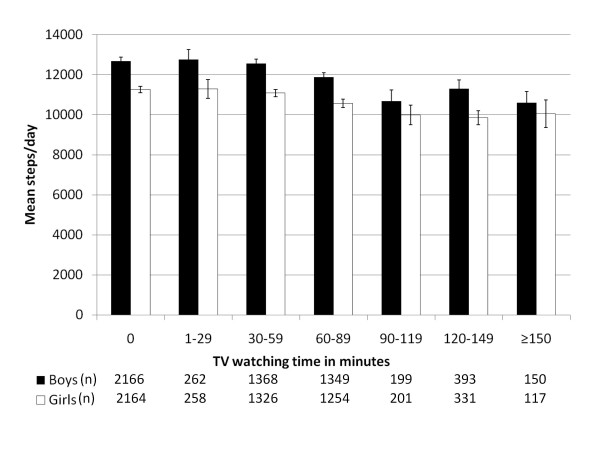
**Mean pedometer-determined steps/day by parent-reported television viewing time on a typical school day, Canadian boys and girls 5-19 years of age**.

Figure 3 displays sex-specific mean steps/day by minutes of television watching between school and dinner. In both boys and girls, a decreasing gradient in steps/day on weekdays (F_6,5885 _= 15.1, p < 0.0001 for boys, F_6,5650 _= 9.6, p < 0.0001 for girls) and across all days of the week (F_6,5896 _= 20.6, p < 0.0001 for boys, F_6,5667 _= 14.4, p < 0.0001 for girls) was evident with increments in television watching behaviour

### Odds of Overweight, Obesity, or Combined Overweight/obesity

Table 2 presents the adjusted odds of being classified as overweight or obese or combined overweight/obese, by steps/day and time spent watching television, adjusted for child's sex, age, and responding parent's education. Overall, 11% of children aged 5-19 years were obese and 17% were overweight. Girls were 23% less likely to be obese and 38% less likely to be overweight than boys. The likelihood of being overweight and obese was lowest among those aged 14-19 years. Children of responding parents who reported at least college education had lower odds of being obese; however, no difference was observed with overweight status. These odds did not differ whether controlling for steps/day or television viewing time or not (data not shown).

**Table 2:** Association between overweight and obesity with pedometer-determined steps/day and parent-reported television watching on a typical school day, Canadian boys and girls 5-19 years

		Average daily steps (weekly)			Average daily steps (weekday)		
		N = 7495			N = 7484		
		Overweight/obese	Overweight	Obese	Overweight/obese	Overweight	Obese
		28%	17%	11%	28%	17%	11%
	%	AOR* (95% CI)	AOR*(95% CI)	AOR* (95% CI)	AOR* (95% CI)	AOR*(95% CI)	AOR* (95% CI)

Male (referent)	51	1.00	1.00	1.00	1.0	1.0	1.0
Female	49	0.66 (0.57, 0.76)	0.62 (0.53, 0.74)	0.77 (0.63, 0.94)	0.66 (0.57, 0.75)	0.62 (0.53, 0.74)	0.76 (0.62, 0.93)

5-9 years (referent)	33	1.00	1.00			1.0	1.0
10-13 years	29	0.56 (0.47, 0.65)	0.95 (0.78, 1.15)	0.25 (0.19, 0.31)	0.56 (0.48, 0.65)	0.95 (0.79, 1.15)	0.25 (0.20, 0.32)
14-19 years	38	0.29 (0.24, 0.35)	0.52 (0.43, 0.65)	0.12 (0.09, 0.16)	0.29 (0.24, 0.35)	0.53 (0.43, 0.65)	0.12 (0.09, 0.16)

< Secondary (referent)	8	1.00	1.00	1.00	1.0	1.0	1.0
Secondary	22	0.96 (0.72, 1.29)	1.07 (0.74, 1.54)	0.85 (0.57, 1.22)	0.98 (0.73, 1.31)	1.08 (0.74, 1.56)	0.86 (0.58, 1.27)
College, trades	33	0.79 (0.60, 1.05)	0.91 (0.64, 1.30)	0.66 (0.45, 0.96)	0.80 (0.60, 1.06)	0.92 (0.64, 1.32)	0.67 (0.47, 0.97)

University	37	0.65 (0.49, 0.86)	0.82 (0.58, 1.17)	0.46 (0.31, 0.67)	0.66 (0.49, 0.87)	0.83 (0.58, 1.19(	0.47 (0.32, 0.69)

per 3000 steps/day	-	0.90 (0.84, 0.95)	0.95 (0.89, 1.01)	0.81 (0.73, 0.89)	0.90 (0.85, 0.95)	0.96 (0.90, 1.03)	0.79 (0.72, 0.87)

per 30 minutes TV	-	1.12 (1.07, 1.17)	1.08 (1.02, 1.14)	1.21 (1.13, 1.29)	1.12 (1.07, 1.18)	1.08 (1.02, 1.14)	1.21 (1.13, 1.30)
Stratified by Age							

5-9 years							
per 3000 steps/day	-	0.87 (0.79, 0.96)	0.98 (0.87, 1.10)	0.77(0.67, 0.89)	0.88 (0.80, 0.96)	1.03 (0.92, 1.15)	0.76 (0.66, 0.86)
per 30 minutes TV	-	1.15 (1.05, 1.26)	1.09 (0.97, 1.22)	1.21 (1.08, 1.36)	1.15 (1.05, 1.26)	1.10 (0.98, 1.23)	1.22 (1.09, 1.36)

10-13 years							
per 3000 steps/day	-	0.87 (0.79, 0.97)	0.89 (0.79, 0.99)	0.81 (0.68, 0.98)	0.87 (0.79, 0.96)	0.89 (0.79, 0.99)	0.80 (0.67, 0.96)
per 30 minutes TV	-	1.13 (1.05, 1.23)	1.09 (1.00, 1.19)	1.24 (1.10, 1.39)	1.13 (1.05, 1.23)	1.09 (1.00, 1.19)	1.23 (1.10, 1.39)

14-19 years							
per 3000 steps/day	-	0.95 (0.86, 1.05)	0.97 (0.88, 1.08)	0.89 (0.74, 1.07)	0.95 (0.86, 1.05)	0.98 (0.88, 1.09)	0.88 (0.74, 1.04)
per 30 minutes TV	-	1.09 (1.02, 1.18)	1.05 (0.97, 1.14)	1.19 (1.06, 1.34)	1.09 (1.02, 1.18)	1.05 (0.96, 1.14)	1.19 (1.05, 1.34)

For every 3000 step increase in weekday pedometer-determined steps/day (equivalent to 30 minutes of moderate-to-vigorous activity in adults [19,20]), the odds of being obese decreased by 21%, and of being overweight/obese by 10%. For every 30 minute increase in time spent watching television between school and dinner on a typical school day, the odds of being obese increased by 20%, of being overweight by 8% and of being overweight/obese by 12%. These relationships were particularly evident among children 5-9 and 10-13 years, whereas for teenagers aged 14-19 years, the relationship between daily steps and obesity was not significant. No interaction effect was observed between steps/day and television watching time among Canadian children aged 5-19 years. Similar results were observed whether considering pedometer-determined steps across all days or weekdays only.

## Discussion

CANPLAY effectively demonstrates that pedometry methods are feasible for obtaining large scale national prevalence data of physical activity levels in children and youth. In fact, CANPLAY is currently the largest database of pedometer data collected in any single sample to date [18]. Similar to findings from other pedometer-based studies [9], Canadian girls take consistently fewer steps/day than their male counterparts. Although, as depicted in Figure 2, steps/day followed a similar overall decreasing pattern with age as that based on smaller and typically school-based samples [18], comparatively, Canadian boys and girls generally took fewer steps/day. In addition, the assembled values [18] portray a distinct increase in pedometer-determined physical activity in childhood (with boys increasing even more than girls), that subsequently declines in youth. In contrast, the CANPLAY data display no notable rise in steps/day in childhood past age 6, but instead depict a slow and steady decline in values through childhood and into youth.

On average, CANPLAY parents reported that Canadian boys and girls watched < 40 minutes of television between school and dinner on a typical school day. Although not directly comparable in terms of the exact time frame queried, ≅36% U.S. youth (aged 14-18 years) in the Youth Risk Behaviour Factor Survey (YRBFS) reported watching ≤ 1 hour television on school days [21] when queried directly. Similarly, ≅39% of U.S. children and youth aged 8-16 years reported that they watched ≤ 1 hour of television on the day prior to their interview conducted as part of the National Health and Nutrition Examination Survey (NHANES); 26% reported watching ≥ 4 hours [22]. We are unable to do more direct comparisons with these examples of American data because of the difference in the time period examined, and the manner in which the question was queried (parent proxy). In addition, the 95^th ^percentile of the distribution of the CANPLAY data was 2 hours and the 99^th ^percentile was 4 hours. It is difficult to say to what degree these discrepancies merely reflect methodological differences or may represent some true country differences in young people's behaviours.

Television watching is only one of many frequently occurring sedentary behaviours in which young people may engage during leisure time. Others include a range of electronic media use such as computer use, video games, social networking, etc. However, television watching has a stronger relationship with pedometer-determined physical activity than video gaming, and consideration of both simultaneously does not necessarily strengthen the relationship in children [14]. Almost half of daily pedometer-determined steps are attributable to after-school activities [23]. If these are pre-empted in favour of television viewing, then an important opportunity to be active is logically lost. Specifically, since time is finite, each hour spent watching television represents a missed opportunity to accumulate up to approximately 6000 steps or more [19]. In addition to replacing opportunities for active alternative behaviours, exposure to television might also impact body weight by promoting excess energy intake. Specifically, evidence indicates that children's television viewing and snacking behaviours are related [24], more television viewing is associated with adverse dietary practices [25], and children consume more available snacks when exposed to food advertising [26]. These additional factors make television viewing potentially more insidious a risk factor for childhood obesity than other forms of sedentary behaviour that are less associated with energy intake.

The odds of being obese were 64% lower for a child in the highest quintile of pedometer-determined physical activity on weekdays (i.e., taking 15075 or more steps/day) than a child in the lowest quintile (i.e., 8664 or fewer steps/day). In contrast, the adjusted odds were 42% higher for a child in the highest quintile of television watching (60 minutes) than another child in the lowest quintile (watching no television between school and dinner). Watching television between school and dinner increases the likelihood of being overweight or obese, and taking more steps/day reduces the likelihood of obesity (but not overweight). Considered together, a child taking ≅15,000 steps/day on weekdays and watching 2 hours of television between school and dinner has a 51% lower likelihood of being overweight/obese and has a 83% lower likelihood of being obese compared to a child taking ≅8780 steps/day and watching no television between school and dinner on a typical school day.

In contrast to the obvious findings related to obesity, a clear association with pedometer-determined physical activity was not apparent with overweight. Although the observed decrease in steps/day and increase in television watching behaviour with increasing BMI-defined obesity categories follows expected changes for the most part, the discrepancy in daily steps is greatest in the transition between the overweight and the obese categories. Stated another way, the overweight children are more like the healthy weight children in their step-defined physical activity behaviours, but differ in their television viewing behaviours. As these are cross-sectional data, we are unable to conclude causal relationships in terms of whether such behaviours caused the overt obesity, or, alternatively, whether they may only reflect current behavioural choices of overweight and obese children in Canada.

A number of limitations must be acknowledged. We only asked the education of the responding parent and relied on parent-reported estimates of children's height and weight. We also only queried about television watching between school and dinner; the effects of television watching at other times was not examined. Pedometers do not detect all types of physical activity, for example swimming, a popular activity among Canadian youth (44%) [27]. However, CANPLAY participants were instructed to remove the pedometer (for example, when swimming) and to record all reasons for removing it during the course of the day. Less than 0.5% reported removing it for swimming during the 7 day period [15]. And although pedometers lack the ability to distinguish time spent in sedentary and low-, moderate- and vigorous-intensity activities, they nonetheless provide valuable information on the total level of physical activity relevant to policy. For example, we found that obesity was inversely associated with pedometer-determined steps/day.

Although it remains plausible that certain individuals may alter their behaviour in response to being knowingly monitored, as previously documented, there was no detectable evidence of reactivity on a population basis in CANPLAY [15]. Furthermore, although additional days provided improved reliability and validity, the first day alone was a reasonable representation of mean steps/day over a 7 day period in terms of reliability (ICC = 0.79) and validity (relative absolute percent error = 2.5%) [15]. Moreover, it is reasonable to presume that the cost of pedometer monitoring is less than that required to conduct accelerometer-based surveillance of a sample of the same size. The CANPLAY pedometers were purchased for $20 CAD each, mailed out and also returned by mail, and data management focused on 7 steps/day entries recorded on a log. In contrast, the NHANES used an accelerometer [28] that cost $335 USD and required initialization, primary distribution was conducted in a face-to-face manner but then return was by mail, and data management required technical expertise and time to download and manipulate manifold amounts of raw data to obtain desired outputs. Finally, we used a 3000 step conversion for 30 minutes of moderate-to-vigorous intensity activity based on adult studies [19,20]. A single study [29] with 10-12 year old children suggests that 3600 steps might be more precise, at least in this age group. *Post hoc *we re-ran the analysis using the 3600 step conversion and found minor (1-2%) decreases in odd ratios for the association between overweight and obesity and pedometer-determined steps/day, but no changes in significance of any of these findings. We decided to stay with presenting the results using the 3000 step conversion as it is a more conservative estimate of the number of steps that a broad age range of children might accumulate by replacing 30 minutes of television watching with activity of various intensities.

Telephone recruitment in CANPLAY permitted the sample design to be less complex than would be required for clustered school-based samples. While this permitted broader coverage, extending to less populated areas, it may have contributed to a higher rate of loss of pedometers (30%) relative to what might be expected in a more controlled environment such as school-based collection (although we know of no comparable published data documenting instrument loss specifically). As we have previously published [15], about 60% of CANPLAY participants returned pedometer data (10% returned pedometers without logged steps) and over 95% of those wore the pedometer for at least 5 days. Participation was lower among 15-19 year olds, those whose parents had less than secondary education, and higher among children age 5-10 years and those whose parents reported a university education [15].

## Conclusions

In summary, CANPLAY is the first large nationally representative sample of young people's pedometer-determined steps/day. It effectively demonstrated reduced odds of being obese or overweight/obese related to each 3000 step increment in physical activity (i.e., 3000 steps/day) whereas each 30 minutes spent watching television (parent-reported) between school and dinner increased the odds of both obesity and overweight status. As a surveillance strategy, the results of CANPLAY can and should inform population obesity approaches including policy schemes.

## Competing interests

The authors declare that they have no competing interests.

## Authors' contributions

CLC and CC monitored original data collection. All authors conceived and designed the present analysis. CLC lead the analysis and results presentation with contributions from CC and JG. CTL and CLC lead interpretation of data with contributions from CC and JG. CTL and CLC led the writing however all authors critically reviewed and approved the final manuscript.

## References

[B1] Olds TS Tomkinson GR Ferrar KE Maher CA Trends in the prevalence of childhood overweight and obesity in Australia between 1985 and 2008 Int J Obes (Lond) 2009 34 57 66 10.1038/ijo.2009.211 19823187

[B2] Ogden CL Carroll MD Curtin LR McDowell MA Tabak CJ Flegal KM Prevalence of overweight and obesity in the United States, 1999-2004 JAMA 2006 295 1549 1555 10.1001/jama.295.13.1549 16595758

[B3] Shields M Overweight and obesity among children and youth Health Rep 2006 17 27 42 16981484

[B4] Tremblay MS Katzmarzyk PT Willms JD Temporal trends in overweight and obesity in Canada, 1981-1996 Int J Obes Relat Metab Disord 2002 26 538 543 10.1038/sj.ijo.0801923 12075581

[B5] Lindsay AC Sussner KM Kim J Gortmaker S The role of parents in preventing childhood obesity Future Child 2006 16 169 186 10.1353/foc.2006.0006 16532663

[B6] Kerr J Norman GJ Sallis JF Patrick K Exercise AIDS, neighborhood safety, and physical activity in adolescents and parents Med Sci Sports Exerc 2008 40 1244 1248 10.1249/MSS.0b013e31816b8797 18580403

[B7] Nielsen reports television tuning remains at record levels digital video recorders grow in popularity http://www.nielsen.com/us/en/insights/press-room/2007/Nielsen_Reports_Television_Tuning_Remains_at_Record_Levels.html

[B8] Tudor-Locke C Pangrazi RP Corbin CB Rutherford WJ Vincent SD Raustorp A Tomson LM Cuddihy TF BMI-referenced standards for recommended pedometer-determined steps/day in children Prev Med 2004 38 857 864 10.1016/j.ypmed.2003.12.018 15193909

[B9] Tudor-Locke C McClain JJ Hart TL Sisson SB Washington TL Pedometry methods for assessing free-living youth Res Q Exerc Sport 2009 80 175 184 19650382 10.1080/02701367.2009.10599551

[B10] Tremblay MS Willms JD Is the Canadian childhood obesity epidemic related to physical inactivity? Int J Obes Relat Metab Disord 2003 27 1100 1105 10.1038/sj.ijo.0802376 12917717

[B11] Russ SA Larson K Franke TM Halfon N Associations between media use and health in US children Acad Pediatr 2009 9 300 306 10.1016/j.acap.2009.04.006 19592321

[B12] Van Der Horst K Paw MJ Twisk JW Van Mechelen W A brief review on correlates of physical activity and sedentariness in youth Med Sci Sports Exerc 2007 39 1241 1250 10.1249/mss.0b013e318059bf35 17762356

[B13] Sallis JF Prochaska JJ Taylor WC A review of correlates of physical activity of children and adolescents Med Sci Sports Exerc 2000 32 963 975 10795788 10.1097/00005768-200005000-00014

[B14] Laurson KR Eisenmann JC Welk GJ Wickel EE Gentile DA Walsh DA Combined influence of physical activity and screen time recommendations on childhood overweight J Pediatr 2008 153 209 214 10.1016/j.jpeds.2008.02.042 18534231

[B15] Craig CL Tudor-Locke C Cragg S Cameron C Process and Treatment of Pedometer Data Collection for Youth: The CANPLAY Study Med Sci Sports Exerc 2010 42 430 435 19952820 10.1249/MSS.0b013e3181b67544

[B16] Cole TJ Bellizzi MC Flegal KM Dietz WH Establishing a standard definition for child overweight and obesity worldwide: international survey BMJ 2000 320 1240 1243 10.1136/bmj.320.7244.1240 10797032 PMC27365

[B17] WHO Consultation on Obesity Obesity: Preventing and Managing the Global Epidemic: Report of a WHO Consultation Geneva, Switzerland 2000 11234459

[B18] Tudor-Locke C McClain JJ Hart TL Sisson SB Washington TL Expected values for pedometer-determined physical activity in youth Res Q Exerc Sport 2009 80 164 174 19650381 10.1080/02701367.2009.10599550

[B19] Tudor-Locke C Sisson SB Collova T Lee SM Swan PD Pedometer-determined step count guidelines for classifying walking intensity in a young ostensibly healthy population Can J Appl Physiol 2005 30 666 676 10.1139/h05-147 16485518

[B20] Marshall SJ Levy SS Tudor-Locke CE Kolkhorst FW Wooten KM Ji M Macera CA Ainsworth BE Translating physical activity recommendations into a pedometer-based step goal: 3000 steps in 30 minutes Am J Prev Med 2009 36 410 415 10.1016/j.amepre.2009.01.021 19362695

[B21] Eisenmann JC Bartee RT Wang MQ Physical activity, TV viewing, and weight in U.S. youth: 1999 Youth Risk Behavior Survey Obes Res 2002 10 379 385 10.1038/oby.2002.52 12006637

[B22] Andersen RE Crespo CJ Bartlett SJ Cheskin LJ Pratt M Relationship of physical activity and television watching with body weight and level of fatness among children: results from the Third National Health and Nutrition Examination Survey JAMA 1998 279 938 942 10.1001/jama.279.12.938 9544768

[B23] Tudor-Locke C Lee SM Morgan CF Beighle A Pangrazi RP Children's pedometer-determined physical activity during the segmented school day Med Sci Sports Exerc 2006 38 1732 1738 10.1249/01.mss.0000230212.55119.98 17019294

[B24] Vader AM Walters ST Harris TR Hoelscher DM Television viewing and snacking behaviors of fourth- and eighth-grade schoolchildren in Texas Prev Chronic Dis 2009 6 A89 19527590 PMC2722406

[B25] Miller SA Taveras EM Rifas-Shiman SL Gillman MW Association between television viewing and poor diet quality in young children Int J Pediatr Obes 2008 1 9 10.1080/17477160801915935 PMC4249761 19086298

[B26] Harris JL Bargh JA Brownell KD Priming effects of television food advertising on eating behavior Health Psychol 2009 28 404 413 19594263 10.1037/a0014399 PMC2743554

[B27] Cameron C Wolfe R Craig CL Physical activity and sport: Encouraging children to be active 2007 Ottawa, ON: Canadian Fitness and Lifestyle Research Institute 120

[B28] Troiano RP Berrigan D Dodd KW Masse LC Tilert T McDowell M Physical activity in the United States measured by accelerometer Med Sci Sports Exerc 2008 40 181 188 18091006 10.1249/mss.0b013e31815a51b3

[B29] Graser SV Pangrazi RP Vincent WJ Step It Up: Activity Intensity Using Pedometers JOPERD 2009 80 22 24

